# Enhancing Analytical Performance of Ammonium Potentiometric Sensors with Carbon Nanocomposites

**DOI:** 10.3390/molecules31050759

**Published:** 2026-02-24

**Authors:** Klaudia Morawska, Szymon Malinowski, Cecylia Wardak

**Affiliations:** 1 Department of Analytical Chemistry, Faculty of Chemistry, Institute of Chemical Sciences, University of Maria Curie-Skłodowska, Maria Curie-Sklodowska Sq. 3, 20-031 Lublin, Poland; klaudia.morawska@mail.umcs.pl; 2 Department of Building Materials Engineering and Geoengineering, Faculty of Civil Engineering and Architecture, Lublin University of Technology, Nadbystrzycka St. 40, 20-618 Lublin, Poland; s.malinowski@pollub.pl

**Keywords:** ion-selective electrodes, ammonium, carbon nanocomposite, solid contact, polymeric membrane

## Abstract

**Highlights:**

1. The use of carbon nanocomposites significantly improves the performance of sensors.
2. A simple and cheap all-solid-state ammonium potentiometric sensor was developed.
3. The developed sensor is resistant to changes in measurement conditions.
4. It is suitable for monitoring ammonium in environmental samples.

**Abstract:**

This paper presents a new type of ammonium electrode that shows a significant improvement in analytical performance compared to unmodified electrodes. The aim of the study was to develop electrodes with better electrochemical parameters, achieved by applying a modification in the form of a solid contact layer based on a carbon nanocomposite consisting of carbon nanofibers and multi-walled carbon nanotubes. Measurements were made to evaluate the basic analytical parameters of both unmodified electrodes and those enriched with an intermediate layer in the form of a carbon nanocomposite. The composite-modified electrodes showed an almost ideal theoretical slope value (58.4 mV·dec^−1^), a lower detection limit, and linearity that remained constant over time. Stability tests showed that electrodes with nanocomposites achieved a potential drift that was almost 50 times lower. An improvement in potential reversibility was also achieved. Another important advantage of the modified electrodes is their resistance to external conditions such as light and the presence of O_2_ and CO_2_. In addition, they exhibit selectivity typical for ammonium electrodes. Based on the results obtained, it was found that the multiwalled carbon nanotubes and carbon nanofibers nanocomposite effectively acts as a solid contact layer, which may form the basis for the development of modern, durable, and precise sensors for the determination of ammonium ions in various environments. The determination of NH_4_^+^ ions in soil was conducted with success.

## 1. Introduction

With the development of industry and global progress, not only is our civilization evolving, but in many ways, this is leading to massive destruction and pollution of the ecosystem. As Paracelsus said: “All things are poison, and nothing is without poison; the dosage alone makes it so a thing is not a poison”. This is why monitoring the levels of particular elements and compounds in the soil is so important, as well as to guard ourselves and our surroundings from exposure to excess levels of different compounds. Ammonium ions (NH_4_^+^) are one of the ions to which we should pay particular attention (especially in agricultural areas) [1,2]. Their excess in the environment is particularly harmful to plants—they not only inhibit root growth but also damage leaves, resulting in photosynthesis disorders. Plant homeostasis is severely disrupted, and this negatively affects not only flora but also agriculture, as high cultivation costs lead to poor and inefficient crops [3,4]. Among other things, their excessive levels cause eutrophication of waters, which leads to the destruction of aquatic environments. Additionally, they are highly detrimental to many water organisms, including fish, causing organ damage and osmoregulation disorders [5,6,7]. This phenomenon also has a negative impact on human health, primarily due to an increased risk of neurological diseases, to which the elderly and children are particularly susceptible. The cause is an increased concentration of ammonium ions (NH_4_^+^) in the surrounding environment [1,8]. The official and permissible concentration of NH_4_^+^ ions in drinking water recommended for the European Union is approximately 0.5 mg/L. Therefore, it is important to monitor the NH_4_^+^ content in soil, because it is from there that these ions enter the water.

One of the methods used to monitor NH_4_^+^ ion levels is potentiometry, in which we use sensors—ion-selective electrodes (ISEs). Our team is engaged in the development of new potentiometric sensors with a type of solid contact, which, due to their imperfections resulting from poor transduction and charge transfer (the result of the inefficiency of this process is poor reversibility and stability of the potential), must be modified by introducing a material that exhibits ion-to-electron conductivity [9,10]. So far, the role of transducer media has been played by conductive polymers [11,12,13], carbon materials [14,15,16], nanoparticles of metals and their oxides [17], hybrid materials [18,19], and composite materials [9,20,21,22]. By using materials with the right characteristics, i.e., ionic and electronic conductivity, stability, and chemical resistance, we achieve improved analytical parameters, mainly stability and potential reversibility [23]. These two parameters are improved due to the enhanced charge transfer process, owing to the presence of a material with dual conductivity (electronic and ionic) between the membrane and the internal electrode. To date, various materials have been used in the construction of ammonium electrodes, including conductive polymers, carbon materials, and advanced nanocomposites (two- and even three-component). An example of a conductive polymer is polypyrrole (PPy), which has been used as a solid contact in sensors for detecting ammonium ions—the sensitivity of such an electrode has been defined by a slope of 56.3 mV·dec^−1^ [24]. Among carbon materials, functionalized multi-walled carbon nanotubes (f-MWCNTs) have been used, which, thanks to their structure and active surface, improve charge transport efficiency and electrode sensitivity [25]. Another example is a nanocomposite, such as a 3D graphene oxide–CNT (carbon nanotube) composite (two-component), which combines the properties of graphene and carbon nanotubes [26], as well as the more complex SiO_2_/ZrO_2_/phosphate–NH_4_^+^ nanocomposite; unfortunately, its production is very time-consuming, which limits its practical and economic application [27].

In this paper, we present an ammonium sensor that is not only an economical option but also quite simple and quick to make and use. It does not require sophisticated reagents or procedures, and it is effective, accurate, and exhibits favorable stability and sensitivity. As a modification to ammonium electrodes (a glassy carbon electrode (GCE) is an internal electrode), a carbon composite material (CNC) consisting of multi-walled carbon nanotubes (MWCNTs) and carbon nanofibers (CNFs) was introduced as a mediating layer. A series of tests were carried out on the electrodes prepared in this way to assess whether the introduction of a carbon solid contact is effective, and the suitability of such an electrode for analytical measurements—the determination of ammonium in soil—was also evaluated.

## 2. Results and Discussion

### 2.1. Basic Potentiometric Measurements—Calibration

**Figure 1:**
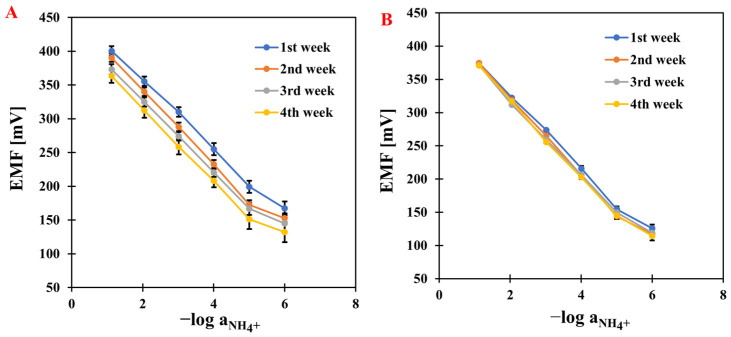
The comparison of electrode response reproducibility based on results obtained for GCE/NH_4_-ISM (**A**) and GCE/CNC/NH_4_-ISM (**B**).

After several days of conditioning the electrodes, measurements were started. The first stage was to determine the ISEs’ response curves. The curves were obtained by plotting the relationship between the electromotive force and the negative logarithm of the main ion (NH_4_^+^) activity. The measurements were carried out over a concentration range of 1 × 10^−7^ to 1 × 10^−1^ M by adding appropriate volumes of NH_4_Cl solutions at concentrations of 1, 0.1, 0.01, and 0.001 M. Based on the determined calibration curves (Figure 1), the electrodes were characterized by determining the basic parameters: slope, detection limit, and linearity range. The measurements were carried out over four weeks for all modified electrodes and compared with a classic, unmodified ammonium electrode—the results are presented in Table 1. The modified electrodes showed increased sensitivity compared to GCE/NH_4_-ISM. It is worth mentioning that in the first week of testing, the electrode modified with nanocomposite achieved a value close to the theoretical slope (58.4 mV·dec^−1^), and the sensitivity remained stable throughout the analysis. The results obtained with our electrodes outperformed those reported for other NH_4_^+^-ISEs in the literature, which showed slopes of 57.3 mV·dec^−1^ [28], 54 mV·dec^−1^ [29], and 49.6 mV·dec^−1^, while their linearity ranges were comparable to those of our sensors [30]. No significant changes were observed in the linearity range (it remained stable during measurements and was 10^−1^–10^−5^ M) and the detection limit (in the micromolar range). The best-performing electrode developed in this work exhibited a lower limit of detection (LOD) of 2.5–3.9 × 10^−6^ M, which is superior to that reported for the NH_4_^+^-ISE described in the literature. In the latter case, accurate determination of NH_4_^+^ ions was not possible at concentrations below its LOD, which is higher than 5.5 × 10^−6^ M [31]. However, in the study reported in [32], a sensor selective to ammonium was demonstrated to exhibit a slightly wider linear range and a marginally lower limit of detection compared to the electrode presented in our work. An extremely impressive improvement was noted in long-term stability, determined as the standard deviation of the E_0_ value over four weeks. The lowest SD was obtained for GCE/CNC/NH_4_-ISM, which was 3.3 mV, indicating that the presence of the composite material significantly improved the stability of the electrode.

### 2.2. Influence of Applied Intermediate Layers on the Potential Stability

**Figure 2:**
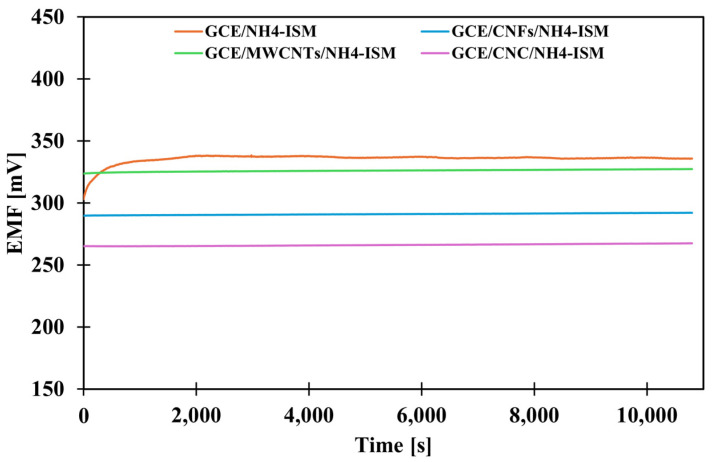
The potential stability of the tested electrodes.

The next stage of the research was to determine how the presence of the various intermediate layers affects the stability of the sensor potential. According to theoretical considerations, the presence of a solid contact layer (intermediate layer) should improve signal stability and minimize potential drift. In order to evaluate how much the proposed modifications affect this parameter, the potential was measured in a solution of the main ion (NH_4_Cl) at a concentration of 1 × 10^−3^ M for three hours. Based on the obtained data, a graph showing the dependence of the potential on time was prepared (Figure 2), and the value of the potential drift was calculated as the quotient of the difference between the starting and ending potentials and the measurement duration (Table 2). Analysis of the results indicates that the most effective modification strategy is to use a composite transducer media as the mediating layer, confirming that composites often exhibit higher efficiency than their individual components. For the CNC-modified electrode, a small potential drift of 0.48 mV·h^−1^ was observed, representing an almost 23-fold improvement in stability compared to the unmodified electrode (10.95 mV·h^−1^), which is a remarkable result.

### 2.3. The Impact of the Solid Contact Material on the Reversibility of the Potential

**Figure 3:**
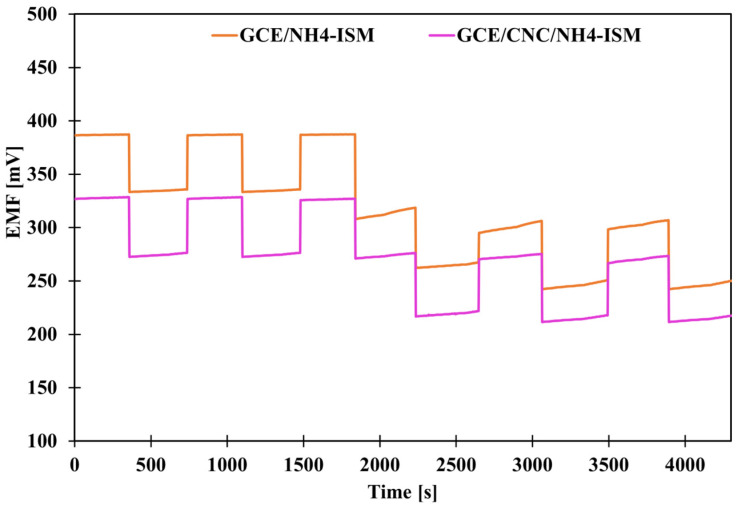
Graphical presentation of potential reversibility for GCE/NH_4_-ISM and GCE/CNC/NH_4_-ISM.

As in the case of potential stability, we also wanted to check the benefit of introducing the proposed intermediate layer, this time by determining the parameter of potential reversibility. Potential reversibility is a fairly important parameter, which was determined by measuring the potential in solutions of different concentrations alternately. The measurements were carried out for about 5 min in each NH_4_Cl solution—first, the measurements were alternated in solutions with concentrations of 10^−4^ and 10^−3^ and then in solutions of 10^−3^ and 10^−2^ M. A graphical illustration of the determined dependence of potential on time under given concentration conditions is shown in Figure 3. Based on the achieved results, deviations from the average potential values at a given concentration were calculated (Table 3). Based on the obtained data, we can conclude that the smallest deviations were recorded for high concentrations, which is a logical conclusion: the more diluted the solution, the greater the deviations from the mean value. CNFs- and MWCNTs-modified electrodes exhibit very similar potential reversibility, several times better than that of the GCE/NH_4_-ISM electrode. However, the use of single-media transducers is not as effective as the use of a composite (combination of these two elements), because the GCE/CNC/NH_4_-ISM electrode showed the smallest deviations from the average values for a given concentration (SD order for decreasing concentrations): 0.11, 0.18, 1.61 mV. This indicates that the best modification option is to use composites of multi-walled carbon nanotubes and carbon nanofibers. The reversibility results are consistent with the stability of the individual electrodes’ potentials, and their responses at specific concentrations correspond well with the potential values obtained from the calibration curves.

### 2.4. How External Conditions Influence Potential Stability

**Figure 4:**
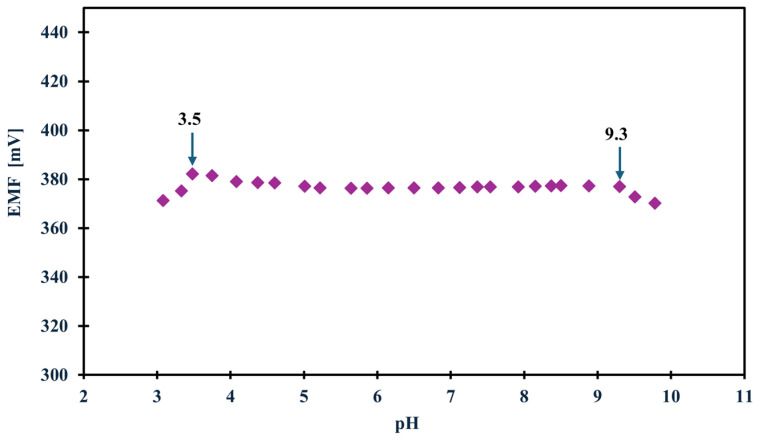
The optimal pH range of the GCE/CNC/ISM electrode.

**Figure 5:**
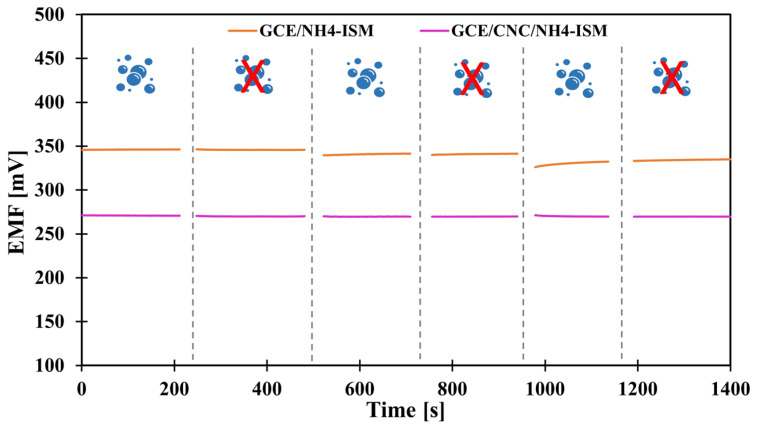
The impact of gases on the electrodes’ potential—results obtained for unmodified electrode and electrode modified with carbon nanocomposite. The shape: 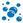
—solution saturated with O_2_ and CO_2_; 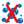
—deoxygenated solution.

In the case of planning field studies or other studies not conducted in laboratory conditions, it is crucial to determine how various external conditions, such as changes in illumination, various sample pH or the influence of sample saturation with different gases, i.e., oxygen, carbon dioxide, and nitrogen, affect the potential (whether it is stable, drifts toward positive or negative values, or perhaps the values shift totally depending on the conditions). Therefore, to determine how the above-mentioned factors affected the tested NH_4_^+^ electrodes, we first carried out measurements in variable lighting conditions. The measurements were carried out alternately in full daylight and in the absence of sunlight in NH_4_Cl samples with a concentration of 1 × 10^−3^ M. None of the tested modified electrodes showed sensitivity to this factor. Only the unmodified electrode showed a potential drift towards higher potential values, which is probably due to the lack of material exhibiting ion-to-electron conductivity rather than sensitivity to light. In turn, the optimal pH range in which the electrode maintained linearity was determined in a 1 × 10^−3^ M KNO_3_ solution, to which additions of nitric acid (V) or sodium hydroxide were made to change the pH of the solution. Based on the data, a curve was plotted showing the dependence of the potential on the pH of the sample—Figure 4 shows the results for the GCE/CNC/ISM electrode. The electrode showed a stable potential between 3.5 and 9.3 pH. The effect of the presence of various gases in the test sample on the electrode potential was also investigated. For this purpose, the potential was measured for several minutes in a 1 × 10^−3^ M NH_4_Cl solution, and there were two sample variants. The first was a classic sample at room temperature saturated by oxygen (O_2_) and carbon dioxide (CO_2_) from the air, while the second sample was deoxygenated by passing a stream of nitrogen (N_2_) through it. The measurements were carried out alternately, and the comparative effects of the obtained results for the unmodified electrode and the one with a carbon composite intermediate layer are presented in Figure 5. The modified electrodes (both with a single-component and two-component solid contact) showed no sensitivity to the presence of gases. For comparison, the unmodified electrode showed slight deviations from the norm, which again makes CNC modification an excellent solution for ammonium electrodes.

### 2.5. Selectivity

Selectivity was analyzed on the basis of potentiometric selectivity coefficients (K_ij_). The measurement was performed using the separate solution method (SSM) and fixed interference method (FIM) for the following interfering ions: K^+^, Na^+^, Li^+^, Ca^2+^, Ni^2+^, Cd^2+^, Zn^2+^, Mg^2+^, Co^2+^, Cu^2+^. The results for each logK_ij_ obtained by the SSM method are shown in Table 4, and those obtained by the FIM method are shown in Table S1. All of the electrodes tested showed typical selectivity, and there were no deviations from the norm. It is worth mentioning that the GCE/CNC/NH_4_-ISM electrode showed the best resistance to the other ISEs for each interfering ion. Based on the data available in the literature [28,33], the selectivity of our sensors is slightly better than that of other electrodes. At the same time, the observed order of decreasing selectivity coefficients for individual ions is consistent in both cases, namely, K^+^, Na^+^, Li^+^, Ca^2+^, Mg^2+^.

### 2.6. Water Layer Test

**Figure 6:**
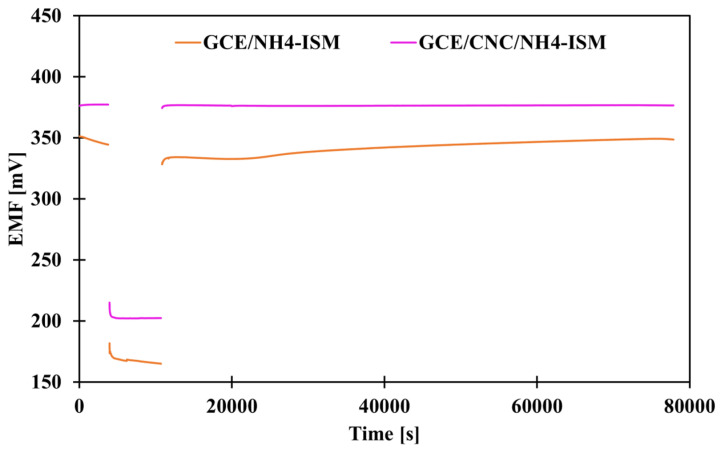
Presentation of water test results for non-modified and composite-modified electrode.

The classic way to determine whether a water layer forms between individual electrode elements, i.e., at the interface between the membrane and the intermediate layer, the membrane and the internal electrode, or the intermediate layer and the internal electrode, which negatively affects the results—their repeatability and reliability—is to perform an appropriate test. This procedure was proposed by Pretsch and co-workers [34]. This involves measuring the potential first in a 0.1 M solution of the main ion (1 h) (NH_4_Cl), then in a 0.1 M solution of the interferent (NaCl) (3 h), and then again in the same solution of the main ion (21 h). When we observe a potential drift after replacing the interfering solution with the main ion, it means that the electrode is unstable and there is a high risk that a water layer has formed between some of the ISE layers. Figure 6 shows a graphical illustration of the results for the control electrode and the electrode modified with carbon nanocomposite. As we can see, the ammonium electrode with a solid contact layer in the form of CNC showed a stable potential after changing the solutions, which proves that it is more resistant to the formation of a water layer than the modified electrode, as such a drift occurred for the GCE/NH_4_-ISM electrode.

### 2.7. Chronopotentiometric Measurements

**Figure 7:**
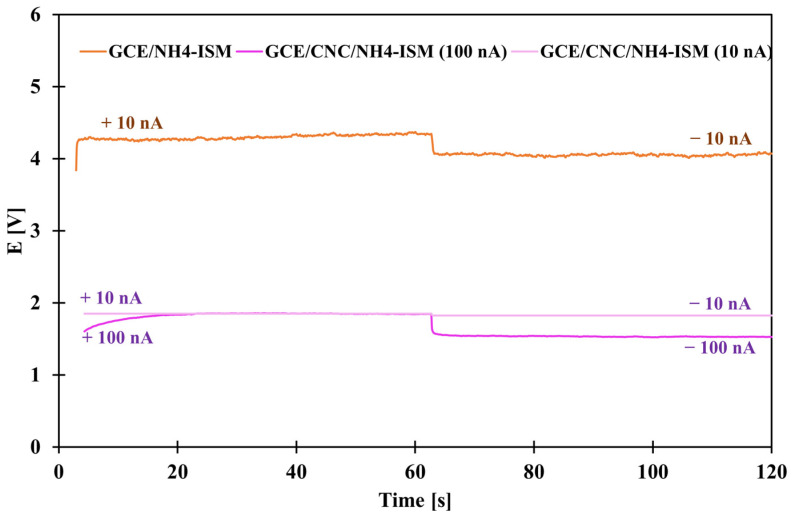
Chronopotentiograms obtained for GCE/NH_4_-ISM (for 10 nA) and GCE/CNC/NH_4_-ISM (for 10 and 100 nA).

Chronopotentiometric studies were conducted to determine the basic electrical parameters. They were performed by applying alternating currents of +10 nA and −10 nA for 60 s (GCE/NH_4_-ISM and GCE/CNC/NH_4_-ISM) and +100 nA and −100 nA for 60 s (GCE/CNC/NH_4_-ISM). Based on the obtained data, the curves shown in Figure 7 were plotted for the unmodified and composite-modified ISEs. Based on the obtained results, the potential drift (defined as the slope of the linear section of the curve) was derived. The electrical capacity (C) was also determined: C = I/(dE/dt), where I is the constant value of the applied current, here 100 and 10 nA, and dE/dt is the linear slope of the chronopotentiometric curve. In addition, the resistance (R) was determined as follows: R = ∆E/I, where ∆E is the potential jump. All results are presented in Table 5. As we can observe not only visually but also on the basis of the calculated results, for each of the parameters, the GCE/CNC/NH_4_-ISM electrode outperformed the GCE/NH_4_-ISM electrode. For the electrode enriched with an intermediate layer in the form of a nanocomposite, not only was a lower potential drift of 0.043 mV·s^−1^ observed but also a reduced resistance compared to the ISE without constant contact, as well as a more than 40 times greater electrical capacity (232 µF). These results confirm how excellent a solution the use of this nanocomposite is in solid-contact sensors sensitive to ammonium ions.

### 2.8. Analytical Appllication

The electrodes we created met all the requirements for use in measuring ammonium ions in real samples, i.e., soil. The soil was collected and then dried (approximately 2 h at 80 °C) and homogenized using a mortar. The sample was divided into three portions, two of which were enriched with ammonium at concentrations of 20 and 40 mg/kg, respectively. Each sample was then subjected to water extraction using a shaker (15 g of soil per 100 mL of distilled water). The sample was shaken for 24 h. After this time, the sample was filtered through a filter and then subjected to pH and potentiometric measurements. The pH of the tested samples was 7.61, which lies well within the optimal operating pH range of the electrode (3.5–9.3). This indicates that the measurements were performed under suitable conditions, ensuring a stable electrode response and eliminating the risk of signal distortion associated with excessively acidic or alkaline environments. Consequently, no pH adjustment was required before analysis, and the obtained results can be considered reliable with respect to the electrode’s specified working range. The determination was carried out using the multiple standard addition method (Gran’s method) using 0.05 M CH_3_COOLi as the ionic strength buffer. The determined ammonium ion content in the soil was 37.6 ± 1.2 mg·kg^−1^. The recovery was 95.6% and 97.2%.

## 3. Materials and Methods

### 3.1. Materials

Membrane components: nonactin (Sigma Aldrich, Buchs, Switzerland), potassium tetrakis-parachlorophenyl borate (KTpClB) (Sigma-Aldrich, Buchs, Switzerland), poly(vinyl) chloride (PVC) (Sigma Aldrich, Steinheim, Germany), bis(1-butylpentyl) adipate (BBPA) (Fluka, St. Louis, MO, USA). Conductive material components: MWCNTs—length ranging from 3 to 6 μm, an outer diameter of approximately 10 nm with a ±1 nm variation, and an inner diameter of approximately 4.5 nm with a ±0.5 nm variation (Sigma Aldrich, Steinheim, Germany), CNFs—diameter 100 nm, length ranging from 20–200 µm and purity ≥ 99.9% (Sigma Aldrich, Steinheim, Germany), THF (Chempur, Piekary Slaskie, Poland). Solutions used in the tests: NH_4_Cl, NH_4_NO_3_, NaCl, KNO_3_, LiNO_3_, Cu(NO_3_)_2_, Ca(NO_3_)_2_, Zn(NO_3_)_2_, Co(NO_3_)_2_, Mg(NO_3_)_2_, Ni(NO_3_)_2_, Cd(NO_3_)_2_, CH_3_COOLi. Other: Al_2_O_3_, distilled water. All the salts used for the potentiometric and chronopotentiometric measurements were high-purity salts for analysis (Fluka, Steinheim, Germany).

### 3.2. Measurements

The potentiometric measurements were conducted using a system consisting of (1) a working electrode—ammonium-ion-selective electrodes modified with various carbon transducer medias, and (2) a reference electrode (silver/silver chloride electrode with a double junction (6.0750.100, Metrohm, Herisau, Switzerland)). The measurements were conducted using 16-channel Lawson Labs data acquisition system (Malvern, PA, USA). All the measurements were carried out at room temperature (23 °C) and mixed using a magnetic stirrer.

The chronopotentiometric measurements were carried out using a three-electrode system: a working electrode (tested ISEs), a reference electrode (silver/silver chloride electrode with double junction 6.0750.100, Metrohm, Herisau, Switzerland) and an auxiliary electrode (carbon rod). The measurements were conducted in 0.1 M NH_4_Cl solution at room temperature using an electrochemical analyzer AUTOLAB (Eco Chemie, Utrecht, The Netherlands) with the program NOVA 2.2.

A high-resolution Quanta 3D FEG scanning electron microscope (FEI Hillsboro, Hillsboro, OR, USA) was used to create images of carbon intermediate-layer materials on the substrate of electrodes. Samples for SEM analysis were dripped from a solution that was then used to apply layers, and then the solvent was allowed to evaporate. The samples were examined using an ETD detector at an accelerating voltage of 20 kV.

### 3.3. Preparation of Ammonium Solid Contact Ion-Selective Electrodes

#### 3.3.1. Membrane Preparation

The ammonium membrane was created by weighing out the appropriate amounts of individual components and then dissolving them in tetrahydrofuran (0.3 g per 3 mL)—the weight percentage of the membrane mixture components was classified as follows: 3% nonactin, 50 mol % KtPBCl, 30% PVC and 66.14% BBPA. The membrane cocktail was then placed in an ultrasonic bath to homogenize the mixture before applying it to the glassy carbon electrode substrates.

#### 3.3.2. Preparation of Transducer Media Solutions

Three conductive media solutions were prepared. Single-component solutions consisted of multi-walled carbon nanotubes (1 mg·mL^−1^) and carbon nanofibers (1 mg·mL^−1^), and a two-component solution that consisted of the aforementioned components at a concentration of 1 mg·mL^−1^ (MWCNTs and CNFs composite). The solutions were prepared in a very simple manner by weighing the appropriate amount of ion-to-electron material and then dissolving it in THF. The composite material was created by mixing the individual components in a 1:1 ratio.

#### 3.3.3. Complex Preparation of the NH_4_-ISEs

Each of the prepared ion-selective electrodes was subjected to preliminary treatment. The glassy carbon electrodes (diameter of GC: 0.3 cm) were first cleaned—polished on moistened Al_2_O_3_, washed with distilled water, additionally cleaned in an ultrasonic bath, and their inner electrode surface was degreased by soaking them in tetrahydrofuran. Then, only a layer of ion-sensitive membrane was dripped onto the GCE/NH_4_-ISM electrode (it served as a control electrode). The remaining electrodes were respectively coated with previously prepared solutions of conductive medium—MWCNTs (GCE/MWCNTs/NH_4_-ISM), CNFs (GCE/CNTs/NH_4_-ISM), and carbon nanocomposite (CNC) (MWCNTs:CNTs) (GCE/CNC/NH_4_-ISM) in a volume of 15 µL. This volume has been found to be sufficient to form a continuous and homogeneous intermediate layer fully covering the surface of the glassy carbon electrode while avoiding excessive thickness that could negatively affect charge transfer properties. This fabrication protocol has been successfully used in our previous work on similar PVC-membrane-based solid-contact nitrate-selective electrodes [20]. After evaporation of the solvent (THF), an ammonium membrane was applied to the surface of the mediating layer in three steps—3 doses of 50 μL each, every 30 min. This approach ensured the formation of a uniform, mechanically stable, and reproducible PVC-based membrane layer of appropriate thickness, leading to satisfactory analytical performance and long-term stability of the electrodes [11]. The electrodes were left again for the solvent to evaporate (24 h) and then placed in a 10^−3^ M KNO_3_ solution for several days (3 days) for conditioning.

### 3.4. Examination of the Intermediate Layers

#### Structure of the Solid Contact

**Figure 8:**
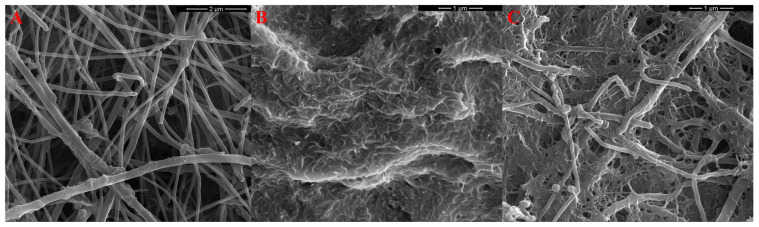
Images of transducer media surfaces: (**A**) carbon nanofiber layer, (**B**) multi-walled carbon nanotube layer, and (**C**) nanocomposite of multi-walled carbon nanotubes and carbon nanofibers.

After applying the layers to the glassy carbon electrode substrate, surface imaging was performed using a scanning electron microscope on both individual composite elements and the composite material. Images showing the surfaces of the applied mediation layers are presented in Figure 8. As we can see, the structures are very clearly visible, and we can also see that carbon nanofibers have been incorporated into the nanotube array, confirming the formation of a nanocomposite.

## 4. Conclusions

A new type of ammonium electrode modified with a nanocomposite of multi-walled carbon nanotubes and carbon nanofibers has been developed. The use of composite material as an intermediate layer has contributed to a significant improvement in the analytical parameters of the electrodes, much better than the use of, for example, a single component of the above-mentioned nanocomposite in this role. The use of CNC contributed to obtaining stable sensitivity over time (58.2–58.3 mV/dec) and potential repeatability, which was verified by determining long-term stability (E^0^)—SD from the E^0^ value was only 3.1 mV. In addition, it was possible to significantly improve the stability (more than 20-fold improvement compared to the control ISE) and reversibility of the potential in comparison to the unmodified electrode. Each of the investigated electrodes exhibited selectivity consistent with that typical for ammonium membranes. Electrical parameters were also measured—we can say with certainty that the introduction of a mediating layer in the form of MWCNTs:CNFs nanocomposite contributed to both an increase in electrical capacity and a reduction in resistance. Thanks to the fact that the electrode passed the water layer test and did not show sensitivity to changes in external conditions, it was successfully used to determine ammonium ions in soil with a recovery close to 100%.

## Figures and Tables

**Table 1:** The basic parameters of ISEs determined at the time of 4 weeks for each electrode.

Parameter	Time	GCE/NH_4_-ISM	GCE/CNFs/NH_4_-ISM	GCE/MWCNTs/NH_4_-ISM	GCE/CNC/NH_4_-ISM
Slope [mV dec^−1^]	1st week	55.8	56.9	57.3	58.4
	4th week	54.3	56.7	57.0	58.2
Detection limit [M]	1st week	6.3 × 10^−6^	5.1 × 10^−6^	4.5 × 10^−6^	3.9 × 10^−6^
	4th week	4.1 × 10^−6^	7.2 × 10^−6^	5.7 × 10^−6^	2.5 × 10^−6^
Linearity range [M]	1st week	10^−1^–10^−5^	10^−1^–10^−5^	10^−1^–10^−5^	10^−1^–10^−5^
	4th week	10^−1^–10^−5^	10^−1^–10^−5^	10^−1^–10^−5^	10^−1^–10^−5^
Long-term stability (E^0^—standard deviation; *n* = 4; *n*_1_ = 1st week, etc.) [mV]	-	452.2 ± 19.6	440.6 ± 9.1	444.1 ± 8.5	438.1 ± 3.3

**Table 2:** Drift of the potential determined for unmodified and modified ISEs.

Parameter	GCE/NH_4_-ISM	GCE/CNFs/NH_4_-ISM	GCE/MWCNTs/NH_4_-ISM	GCE/CNC/NH_4_-ISM
Short-term potential drift [mV·h^−1^]	10.95	0.96	0.81	0.48

**Table 3:** The values of standard deviation determined for each electrode on the basis of mean potential values obtained for different concentrations.

Concentration of Main Ion	GCE/NH_4_-ISM	GCE/CNFs/NH_4_-ISM	GCE/MWCNTs/NH_4_-ISM	GCE/CNC/NH_4_-ISM
1 × 10^−2^ M (*n* = 3)	0.70	0.48	0.39	0.11
1 × 10^−3^ M (*n* = 5)	8.59	2.15	1.34	0.18
1 × 10^−4^ M (*n* = 3)	8.34	2.56	2.02	1.61

**Table 4:** The selectivity coefficients logK_ij_ determined for each electrode for various interfering ions using the separate solution method.

Interfering Ion	GCE/NH_4_-ISM	GCE/CNFs/NH_4_-ISM	GCE/MWCNTs/NH_4_-ISM	GCE/CNC/NH_4_-ISM
K^+^	−1.19	−1.24	−1.30	−1.35
Na^+^	−3.41	−3.48	−3.58	−3.70
Li^+^	−5.46	−5.48	−5.64	−5.77
Ca^2+^	−6.01	−5.95	−6.03	−6.27
Ni^2+^	−0.88	−0.85	−0.88	−1.02
Cd^2+^	−4.39	−4.47	−4.60	−4.69
Zn^2+^	−3.13	−3.22	−3.27	−3.30
Mg^2+^	−6.41	−6.39	−6.56	−6.77
Co^2+^	−2.42	−2.54	−2.58	−2.59
Cu^2+^	−3.45	−2.84	−3.43	−3.47

**Table 5:** The parameters calculated on the basis of chronopotentiometric measurements.

Ion-Selective Electrode	Current Value [nA]	Potential Drift [mV·s^−1^]	Resistance [MΩ]	Electrical Capacitance [µF]
GCE/NH_4_-ISM	10	1.76	155	5.68
GCE/CNC/NH_4_-ISM	10	0.043	2.81	232
	100	0.43		

## Data Availability

The original contributions presented in this study are included in the article. Further inquiries can be directed to the corresponding author.

## Supplementary Materials

The following supporting information can be downloaded at: https://www.mdpi.com/article/10.3390/molecules31050759/s1. Table S1. The selectivity coefficients log Kij determined for each electrode for various interfering ions using the FIM method.

## Abbreviations

The following abbreviations are used in this manuscript:

BBPA	bis(1-butylpentyl) adipate
C	electrical capacitance
CNC	carbon nanocomposite
CNF	carbon nanofiber
E	potential
EMF	electromotive force
GCE	glassy carbon electrode
I	current
ISE	ion-selective electrode
ISM	ion-sensitive membrane
K_ij_	potentiometric selectivity coefficients
KtPBCl	potassium tetrakis-parachlorophenyl borate
MWCNT	multi-walled carbon nanotubes
PPy	polypyrole
PVC	poly(vinyl) chloride
R	resistance
SD	standard deviation
SEM	scanning electron microscopy
THF	tetrahydrofuran
