# Supplementary material for: Enhancing Analytical Performance of Ammonium Potentiometric Sensors with Carbon Nanocomposites
Source: Molecules. 2026 Feb 24;31(5):759. doi: 10.3390/molecules31050759 (PMC12985601; doi:10.3390/molecules31050759)
Supplement: Supplementary file 1 [file molecules-31-00759-s001.zip › molecules-4150963-supplementary.pdf]

Enhancing Analytical Performance of Ammonium Potentiometric Sensors with Carbon Nanocomposites

Klaudia Morawska [1], Szymon Malinowski [2] and Cecylia Wardak [1,*]

[1] University of Maria Curie-Skłodowska, Institute of Chemical Sciences, Faculty of Chemistry, Department of Analytical Chemistry, Maria Curie-Sklodowska Sq. 3, 20-031 Lublin, Poland; cecylia.wardak@mail.umcs.pl; Klaudia.morawska@mail.umcs.pl

[2] Lublin University of Technology, Faculty of Civil Engineering and Architecture, Department of Building Materials Engineering and Geoengineering, Nadbystrzycka St. 40, 20-618 Lublin, Poland; s.malinowski@pollub.pl

* Correspondence: cecylia.wardak@mail.umcs.pl

Table S1. The selectivity coefficients log K_{ij} determined for each electrode for various interfering ions using FIM method.

Interfering ion	GCE/NH_4-ISM	GCE/CNFs/NH_4-ISM	GCE/MWCNTs/NH_4-ISM	GCE/CNC/NH_4-ISM
K^+	-1.19	-1.34	-1.26	-1.35
Na^+	-3.22	-3.59	-3.60	-3.85
Li^+	<-5	<-5	<-5	<-5
Ca^{2+}	<-5	<-5	<-5	<-5
Ni^2	-0.82	-1.05	-0.86	-1.20
Cd^{2+}	-4.56	-4.25	-4.42	-4.50
Zn^{2+}	-3.13	-3.18	-3.45	-3.41
Mg^{2+}	<-5	<-5	<-5	<-5
Co^{2+}	-2.61	-2.54	-2.60	-2.72
Cu^{2+}	-3.40	-2.81	-3.40	-3.50
